# Age-Related Differences in Cognitive and Postural Performance During Dynamic Dual-Tasks

**DOI:** 10.3390/s26061847

**Published:** 2026-03-15

**Authors:** Elisa Misley, Maria Chiara Delatto, Maura Casadio, Tommaso Falchi Delitala, Valeria Falzarano, Giorgia Marchesi

**Affiliations:** 1 Dipartimento di Informatica, Bioingegneria, Robotica e Ingegneria dei Sistemi (DIBRIS), University of Genoa, 16145 Genoa, Italy; maura.casadio@unige.it; 2 Movendo Technology srl, 16149 Genoa, Italy; mariachiara.delatto@movendo.technology (M.C.D.); tommaso.falchi@movendo.technology (T.F.D.); valeria.falzarano@movendo.technology (V.F.)

**Keywords:** dual-task, older adults, postural control, balance perturbations, robotic platform, cognitive functions, balance performance

## Abstract

**Highlights:**

**What are the main findings?**

- Reaction time during executive demanding motor–cognitive dual tasks is highly sensitive to age-related cognitive decline in unimpaired adults.
- Center of pressure and trunk sway areas are the strongest markers of balance decline, especially during dynamic balance under high executive-demand tasks in unimpaired adults.

**What are the implications of the main findings?**

- Executive-demanding motor–cognitive dual tasks combined with dynamic balance provide sensitive tools for early detection of age-related cognitive and balance decline in unimpaired adults.
- These outcomes provide valuable normative references for the early detection and monitoring of age-related cognitive and balance decline and may help to distinguish aging from emerging pathological changes.

**Abstract:**

Age-related declines in balance and cognitive function increase fall risk and reduce quality of life in older adults and people with neurological disorders. Studying these changes in unimpaired adults provides a normative reference for identifying pathological deviations. However, most dual-task studies focus on single cognitive tasks and static conditions, specifically during gait, limiting understanding of how cognitive demand interacts with postural control while standing and during dynamic challenges. This study identified cognitive and motor outcomes most sensitive to age-related differences during motor–cognitive dual tasks of varying complexity across static and dynamic balance conditions, accounting for minimal detectable change. Sixty healthy adults performed dual-tasks ranging from simple motor activities to complex cognitive challenges (Stroop Test) while standing on a robotic platform. Cognitive performance (reaction time) and balance outcomes, including trunk and center of pressure (CoP) sway area, were assessed. Reaction time was sensitive to aging, with standardized estimates ranging from 0.014 to 0.036. The highest values occurred in the most demanding dual-task condition, enabling detection of meaningful change over short timeframes. Age effects on balance were modest under static conditions but amplified during dynamic perturbations across all dual tasks. In the SCWT 3 condition, standardized estimates for CoP sway area increased from 0.006 in the static condition to 0.047 in the passive condition, reflecting an approximately eightfold increase in age sensitivity. Trunk sway primarily reflected cognitive load, whereas CoP sway was most sensitive to balance perturbations and exceeded minimal detectable thresholds over only a couple of years. These findings support sensitive task–condition combinations for early detection and monitoring of age-related cognitive and balance decline.

## 1. Introduction

Motor and cognitive impairments are common in neurological disorders such as Parkinson’s disease, Traumatic Brain Injury, and Multiple Sclerosis, arising from damage to specific brain regions or neural pathways [1,2,3]. Common motor impairments include bradykinesia, rigidity, tremor, spasticity, and impaired coordination [4,5]. These impairments have an adverse effect on the execution of functional tasks, resulting in reduced gait speed, shorter step length, turning difficulties, and postural instability [6,7]. Such limitations substantially increase the risk of falls and contribute to a progressive loss of independence in daily activities [8,9,10]. In addition, cognitive deficits, such as reduced processing speed, memory impairment, attentional difficulties, and impaired executive function, often co-occur and aggravate motor symptoms [11,12]. In particular, executive dysfunction has been consistently associated with reduced balance performance and increased fall risk in individuals with neurological disorders [12,13].

In these populations, symptoms are exacerbated during Motor–Cognitive Dual-Tasks (MCDTs), which involve the simultaneous execution of motor and cognitive activities, such as walking while talking on the phone or driving while listening to a podcast. While unimpaired adults typically manage dual-tasking with relative ease, individuals with neurological impairments often experience performance declines in the motor task, the cognitive task, or both [14,15,16].

Even in unimpaired individuals, however, switching from single-task (ST) to dual-task (DT) conditions induces measurable performance decrements, reflecting the limited capacity of attentional resources. In young adults, the dual-task cost (i.e., the percentage change in performance between dual-task and single-task conditions) is generally small and depends on the relative difficulty and attentional demands of the two tasks, often manifesting as a slight decline in either motor or cognitive performance [17,18].

With aging, this dual-task cost becomes more pronounced. Older adults exhibit greater performance declines under DT conditions due to age-related reductions in executive function, attentional capacity, and sensorimotor efficiency [19,20,21,22]. In the context of balance, they show larger decreases in postural stability, likely because maintaining upright control requires increased cognitive resources [23]. Neuroimaging studies support this notion, demonstrating increased prefrontal cortex activation during balance tasks in older individuals, suggesting a shift from automatic postural strategies toward more consciously controlled mechanisms [24,25,26].

Understanding how dual-tasking affects balance in unimpaired individuals is essential for establishing normative age-related changes and distinguishing physiological aging from pathological deterioration. Evidence indicates that MCDTs can reveal subtle impairments even in unimpaired older adults, making them particularly sensitive tools for early detection. Early identification of these changes is crucial for developing targeted interventions and optimizing rehabilitation strategies [27].

Although MCDTs have received considerable attention, investigations have predominantly focused on gait, often using a single cognitive task as a concurrent distractor, while postural stability has remained relatively neglected. However, investigating postural control during standing is essential, as many daily activities involve maintaining upright stability while concurrently performing cognitive tasks. Unlike gait, standing is characterized by greater sensorimotor cortical connectivity, reflecting increased supraspinal control relative to the more spinal and subcortical control [28]. This organization reflects the need for continuous, precise postural adjustments to maintain upright stability [29,30]. Consequently, analysing postural stability may offer greater sensitivity in detecting subtle balance impairments, particularly in aging populations or individuals with early-stage neurological conditions. Moreover, most research of postural control has focused predominantly on static conditions [24,25], even though dynamic balance tasks, including transitional movements and responses to external perturbations, require different control strategies and more accurately reflect real-world functional demands. Another limitation of current MCDT research is its narrow focus on a single type of cognitive task. This is problematic because research suggests that dual-task effects on balance depend critically on both the nature and the cognitive demands of the concurrent task [31]. Finally, many dual-task studies have traditionally treated the cognitive task as a “distractor”, focusing primarily on motor performance and neglecting cognitive outcomes [32]. This approach overlooks the bidirectional nature of cognitive-motor interference. Examining how balance tasks affect cognitive performance may help uncover subtle cognitive deficits that remain undetected under single-task conditions.

To address these limitations, we conducted a study in which unimpaired participants performed tasks of progressively increasing complexity, ranging from single-task balance exercises to dual-task conditions combining balance with motor tasks and digital cognitive assessments, including reaction time, serial subtraction, and the Stroop Color Word Test [33,34,35]. All tasks were performed while standing on a robotic platform under both static and dynamic balance conditions. This study aimed to identify the cognitive and balance outcomes most sensitive to age-related differences in cognitive and postural performance during dual-task conditions of varying complexity across static and dynamic balance tasks.

## 2. Materials and Methods

### 2.1. Set Up and Protocol

**Figure 1:**
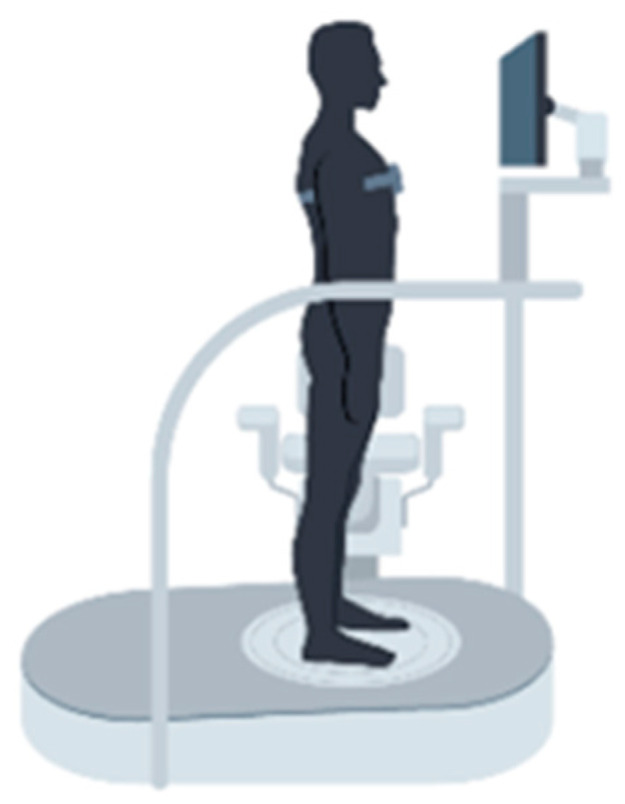
Hunova device used in this study.

This study utilized hunova (Movendo Technology s.r.l., located in Genoa, Italy), a medical robotic device for sensory-motor rehabilitation and assessment of lower limbs and trunk [36]. The system comprises two electromechanical platforms, positioned under the feet and under the seat (as shown in Figure 1). Each platform consists of two motorized axes and includes a custom six-axis force/torque sensor from which we can derive the center of pressure (CoP) measurements. A wireless inertial sensor was placed on the participants’ sternum to track trunk motion. Both the CoP and trunk displacement measurements were computed along the anteroposterior (AP) and mediolateral (ML) planes.

Participants performed the tests while standing in an upright posture, with heels approximately 2 cm apart, feet abducted at 20°, and arms relaxed at their sides, as described in [37].

Three balance conditions were used during the testing:

- Static. The platform remained parallel to the horizontal plane without any movement or tilting, providing a stable surface.
- Unstable. The platform simulated a plate with a central pivot, that tilted due to the user’s weight shift, while an elastic force field, opposing the user’s motion, attracted the platform to the horizontal position.
- Passive. The platform imposed an externally driven continuous perturbation by following a pre-programmed circular trajectory, independent of the participant’s sway [38].

Before testing, participants completed a familiarization phase under all balance conditions. This part allowed them to acclimate to the platform’s movements before actual testing. These postural conditions were performed alone (ST), or simultaneously with a concurrent task (DT).

The DT conditions included the following concurrent tasks:

- Reaction Time Test (RTT): an icon displayed on the screen changed color at random intervals, and participants were instructed to touch it as quickly as possible.
- Serial Subtraction (SS): participants repeatedly subtracted a fixed number from a randomly selected starting number between 90 and 99 (by 7 s in static conditions and by 3 s in dynamic conditions).
- Stroop Color and Word Test (SCWT): Three subtasks were administered, corresponding to the original Word Reading, Color Naming, and Interference conditions. In our digital adaptation [32], each trial presented a stimulus at the center of the screen (a color word in black ink, a sequence of colored Xs, or a color word in incongruent ink), with four answer options displayed as buttons below. Participants indicated their response by reaching and touching the button containing correct answer.

All ST and DT trials lasted 30 s, except for the RTT, whose duration varied (17.6–37.6 s) due to the random presentation of stimuli. For all analyses, the first 5 s of each trial were discarded. Two experimental protocols were designed to avoid participants from repeating the same cognitive task in different balance conditions. Participants were randomly assigned to one of the two protocols (details in Table 1). Within each protocol, tasks were grouped into two blocks, and the order of the tasks within each block was randomized to prevent consecutive repetition of either the same cognitive task or balance condition.

### 2.2. Subjects

A total of 60 unimpaired individuals (aged 18–80 years old) voluntarily participated in this study. The mean age of the participants was 47.9 ± 16.7 years, with 24 males and 36 females. They were divided into two groups, each completing a different assessment protocol: G1—30 subjects, 18 females, age 46.6 ± 16.0 years old—underwent protocol 1; G2—30 subjects, 17 females, age 48.2 ± 16.8 years old—underwent protocol 2. Inclusion criteria required participants to have no neurological disorders or other conditions that could impair balance, to be able to walk independently and to show no signs of cognitive impairment. The study procedures conformed to the Declaration of Helsinki and were approved by the ethical committee of the University of Genoa (protocol no. 2024.06, approved on 31 January 2024). All subjects included in the study signed a consent form that conforms to these guidelines and approved to publish individual data.

### 2.3. Data Analysis

All raw data were processed and analyzed using Python (version 3.11).

#### 2.3.1. Cognitive Performance

To assess cognitive performance, we considered:

- Mean reaction time: reaction time was defined as the interval between stimulus onset and the participant’s response (touch on the screen). For each participant, mean reaction time was computed as the average across all correct trials [39] in both the RTT and the SCWT.
- Number of correct answers: for SS, the total number of correct subtractions, normalized by the maximum possible number of responses for each subtraction task (by 3 s and by 7 s), to ensure comparability across difficulty levels. For SCWT, the total number of correct responses for each test section.

#### 2.3.2. Balance Performance

To assess balance performance, we analysed outcomes derived from the CoP and the inertial sensor data. Such signals were recorded at a sampling frequency of 30 Hz, corresponding to the default sampling frequency of hunova, corresponding to the default sampling frequency of hunova, as already used in previous studies [40,41,42]. Trunk acceleration signals were then reoriented to a true horizontal-vertical Cartesian coordinate system, following the procedure described in [37]. For consistency with previous analysed data, all signals were then filtered with a Butterworth lowpass filter with a cutoff frequency of 7 Hz [37].

The primary measures for this study were:

- Trunk Sway Area (m^2^/s^4^): the 95% confidence ellipse area of trunk accelerations projected in the horizontal plane; this is a measure of the overall magnitude of trunk movement during standing. Higher values indicate greater trunk instability, reflecting reduced postural control, whereas lower values reflect more stable trunk control.
- CoP Sway Area (cm^2^): the 95% confidence ellipse of the statokinesigram of the CoP displacement; this measure reflects the area over which the CoP moves while maintaining balance. Increases in CoP sway area indicate reduced balance stability or greater postural adjustments, while decreases suggest more stable postural control.

In addition, we considered the maximum amplitude of the trunk oscillations (°) and the maximum amplitude of the CoP displacement (cm) in both anteroposterior (AP) and mediolateral (ML) directions as secondary measures.

### 2.4. Statistical Analysis

The aim of this study was to establish which cognitive and balance outcome most sensitively capture age-related differences in both cognitive and postural performance in varying dual-task scenarios in both static and dynamic conditions.

Prior to this main analysis, it was necessary to ensure that participants assigned to the two experimental protocols did not differ systematically in their baseline performance. Baseline equivalence between protocols was verified using a general linear model with protocol as fixed factor and age as covariate. As expected, no significant group differences in balance performance were detected. For completeness, the full results of this analysis are presented in the Supplementary Materials.

Before addressing the main objective, we first sought to gain an overall understanding of how balance condition (static, unstable, passive), task type (balance alone and balance combined with additional concurrent tasks), and age influenced each outcome measure, both individually and through their interactions. We therefore fitted a comprehensive mixed-effects model, using Jamovi software (version 2.6.44), for each balance and cognitive outcome, with balance condition and task type as fixed effects, age and task duration as continuous covariates, and participant (subject ID) as a random factor to account for both repeated observations within individuals and unequal numbers of trials across participants. The task duration was included as a covariate only for balance outcomes. More specifically, for cognitive metrics, the task type comprised the RTT and SCWT 1–3 for the reaction time, while it comprised the SS and SCWT 1–3 tasks for the number of correct answers. For balance metrics, task type included both ST and all the MCDTs presented in this study. The raw data were not normally distributed, but this did not pose a problem, as linear mixed-effects models do not assume normality. Instead, the normality of the residuals was assessed through visual inspection of Q–Q plots. Statistical significance was set at *p* < 0.05 for all analyses.

To achieve our main objective, we simplified the previously described mixed-effects model, which had provided an overall understanding of how our factors influenced both balance and cognitive performance. We did so by analyzing each balance condition separately, thus removing balance condition as a factor from the model. For both balance and cognitive outcomes, this simplified mixed-effects model included task type as a fixed effect, age and task duration (only for balance outcomes) as continuous covariates, and participant as a random factor. This model allowed us to focus on the age*task type interaction within each balance condition. For outcomes where this interaction was significant, we computed the standardized estimates to quantify the magnitude of age-related effects for each task type. The standardized estimates were computed following [43] and expressed as standard deviation units of the dependent variable per one-year increase in age, enabling direct comparison of age-related effects across all balance outcomes on a common scale. Additionally, we calculated the annual change for each outcome to quantify how much each variable increased or decreased over one year. The annual change was computed by multiplying the standardized estimates by the standard deviation of each outcome variable, converting the effect into the original measurement units. Although these estimates are not traditional effect sizes, they were used to characterize and interpret the magnitude of age-related effects. All post hoc pairwise comparisons were adjusted using the Bonferroni correction to control for multiple testing.

## 3. Results

All participants completed all the proposed exercises without experiencing fatigue.

Each factor—task type, balance condition, and age—significantly influenced both cognitive and balance performance, as detailed in the following sections.

### 3.1. Cognitive Performance

**Figure 2:**
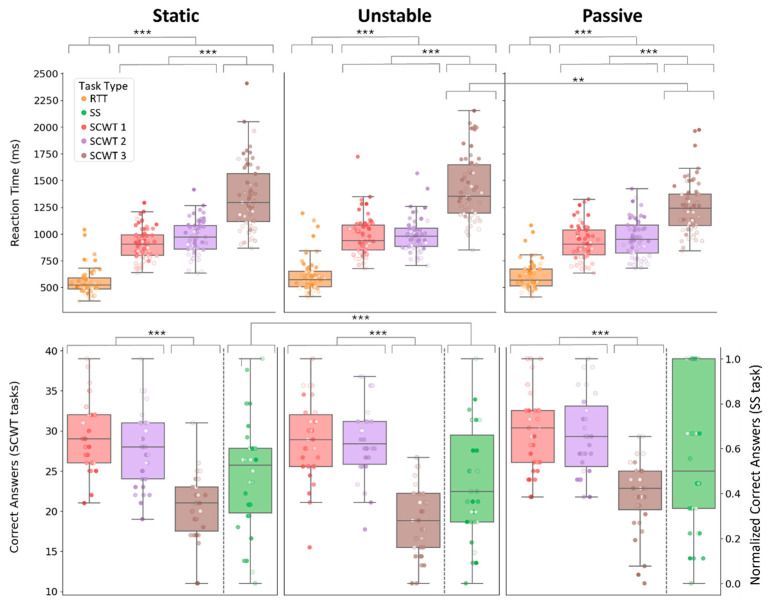
Reaction times (**top row**) and number of correct answers (**bottom row**) are shown across task type and balance conditions. Reaction times were assessed for RTT and SCWT 1–3, while the number of correct answers was examined for SCWT 1–3 and SS, under Static, Unstable, and Passive platform conditions. The left y-axis in the bottom row represents the number of correct answers for the SCTW, while the right y-axis is used exclusively for the SS task, for which the number of correct answers is normalized. Scatter points represent individual data, with color encoding participant age: darker hues indicate older participants, and lighter hues indicate younger participants. Significance levels are indicated as follows: *p* < 0.01 (**), and *p* < 0.001 (***).

Results for the mixed-effects models tailored to each cognitive outcome are reported in Table 2. Both reaction time and number of correct answers showed significant main effects of balance condition (*p* = 0.019, *p* < 0.001), task type (both *p* < 0.001), and age (*p* = 0.001, *p* < 0.001). Increasing the complexity of the concurrent task resulted in longer reaction times and fewer correct responses. Specifically, as shown in the top panel of Figure 2, reaction times were significantly longer in all SCWTs compared to RTT (all *p* < 0.001), and SCWT 3 elicited significantly slower responses than SCWT 1 and 2 (both *p* < 0.001), whereas SCWT 1 and 2 did not differ from each other. Similarly, as shown in the bottom panel of Figure 2, the number of correct responses was comparable between SCWT 1 and 2 but decreased significantly in SCWT 3 (*p* < 0.001). Also, longer reaction times and fewer correct responses were observed with increasing age (all *p* ≤ 0.001). Balance condition also influenced cognitive performance (reaction time: *p* = 0.019, correct answer: *p* < 0.001, Table 2), with unstable condition yielding the poorest cognitive performance compared to both static and passive scenarios. Specifically, reaction times were longer in the unstable condition than in both static and passive conditions, although only the unstable–passive comparison was significant (*p* = 0.016; unstable–static: *p* = 0.070). The number of correct answers was also significantly reduced in the unstable condition; however, significance was observed only for the static–unstable comparison (*p* < 0.001).

Significant balance*task-type interactions were found for both correct responses (*p* = 0.004) and reaction times (*p* = 0.004). Nonetheless, post hoc analyses indicated a generally consistent pattern across balance conditions for both cognitive outcomes: reaction times increased progressively from RTT to SCWT 3, and the number of correct responses decreased, with SCWT 3 showing the lowest performance among all SCWTs (Figure 2). The interaction effect for reaction time was primarily driven by SCWT 3, which showed condition-specific differences. Participants showed the longest reaction times in the unstable condition, followed by the static condition, with the shortest reaction times in the passive condition. The difference reached statistical significance only between the unstable and passive conditions (*p* = 0.005). For correct responses, the SS task largely accounted for the significant interaction, as its performance was clearly influenced by the balance condition. The number of correct responses was lowest in the unstable condition, with significantly fewer correct responses than in the static condition (*p* < 0.001).

In contrast, the balance*age interaction was never significant for any cognitive outcome (*p* > 0.050), indicating that the effect of age on cognitive performance was consistent across balance conditions. However, age interacted with task type (all *p* < 0.001). The three-way interaction (balance*age*task-type) did not reach significance; however, our primary hypothesis was to identify which combination of task type and balance condition most effectively reveals age-related differences. Therefore, we performed planned follow-up analyses examining the age*task-type interaction within each balance condition. Results are reported in Table 3.

Significant age*task-type interactions were observed in all balance conditions for reaction time (all *p* < 0.001). However, it was only under dynamic conditions that the number of correct answers was significant (unstable: *p* = 0.002, passive: *p* = 0.010, see Table 3).

For reaction time, standardized estimates increased with the complexity of the concurrent task, with comparable values across balance conditions: estimates were smallest for the RTT, increased for SCWT 1 and 2, and were highest for SCWT 3 (Table 3 for details). In the RTT, age-related changes reached significance only in the unstable condition, while for SCWT 1–3, they were significant across all balance conditions. The largest effect was observed in SCWT 3 in static condition. The estimated annual increase in reaction time ranged from approximately 1.39 ms in the RTT (unstable condition) to about 11.30 ms in SCWT 3 (static condition).

For the number of correct answers, age-related declines were observed only in the unstable and passive conditions. No significant age-related differences emerged for the SS task in either condition (both *p* > 0.05). In contrast, all SCWT tasks showed significant decreases (Table 3). To contextualize these effects, the estimated annual reduction in the number of correct responses ranged from approximately 0.08 to 0.17 across SCWT tasks. All annual changes are reported in the Supplementary Materials.

### 3.2. Balance Outcomes

**Figure 3:**
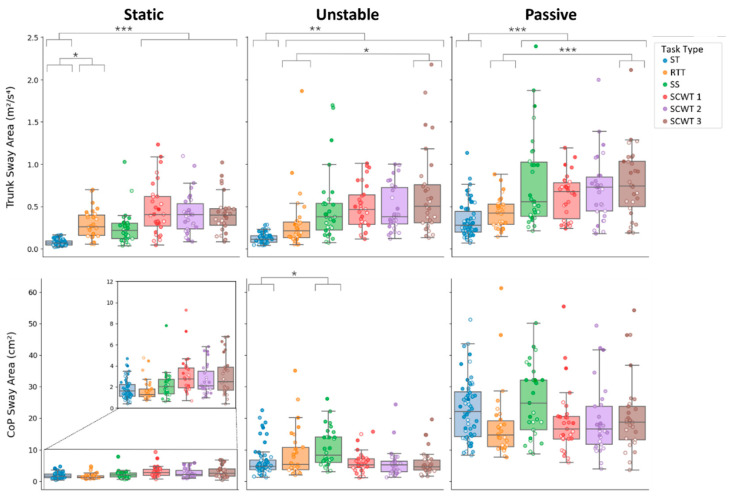
Trunk sway area (**top**) and CoP sway area (**bottom**) are shown across task type and balance conditions. The boxplots show the distribution of sway metrics for all the task performed (ST, RTT, SS, SCWT 1, SCWT 2, SCWT 3) during Static, Unstable, and Passive platform conditions. For the Static CoP condition, a zoomed inset highlights differences in lower-range sway values that are not visible on the full-scale plot. A color gradient is used for the scatter points, with lighter hues indicating younger participants and darker hues indicating older participants. Significance levels are indicated as follows: *p* < 0.05 (*), *p* < 0.01 (**), and *p* < 0.001 (***).

**Figure 4:**
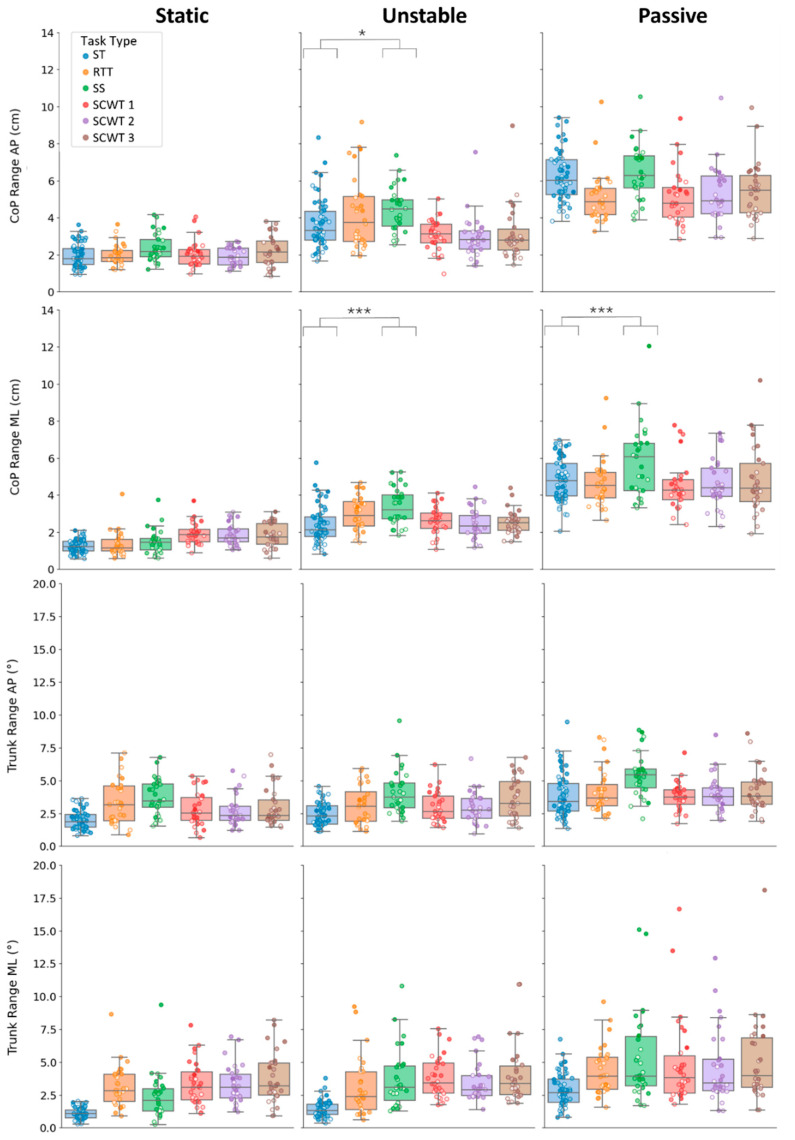
CoP ranges (**2 top rows**) and trunk ranges (**2 bottom rows**) are shown across task type and balance conditions. The boxplots show the distribution of CoP and trunk range metrics for all the task performed (ST, RTT, SS, SCWT 1, SCWT 2, SCWT 3) during Static, Unstable, and Passive platform conditions. A color gradient is used for the scatter points, with lighter hues indicating younger participants and darker hues indicating older participants. Significance levels are indicated as follows: *p* < 0.05 (*), and *p* < 0.001 (***).

Across all balance outcomes, balance condition, task type, and age showed robust main effects (all *p* ≤ 0.001), as shown in Table 4. More challenging balance conditions and more cognitively demanding tasks were associated with worst balance performance, as indicated by increased sway in both trunk and CoP measures (Figure 3 and Figure 4). In addition, aging was associated with a general decline in balance performance.

For primary outcomes, significant balance*task-type and balance*age interactions were observed, indicating that the impact of task complexity and age on both trunk and CoP sway areas varied across balance conditions. In contrast, these interactions were significant only for CoP measures among the secondary outcomes, suggesting that task complexity and age influenced CoP ranges but not trunk ranges across balance conditions (Table 4).

Focusing on the significant balance*age interactions found for primary outcomes and CoP ranges (all *p* < 0.001): age-related increases in sway were systematically larger in dynamic than static conditions, as indicated by the coefficients for trunk area (static: Est = 0.005, *p* = 0.148; unstable: Est = 0.021, *p* < 0.001; passive: Est = 0.018, *p* < 0.001), CoP area (static: Est = 0.004, *p* = 0.203; unstable: Est = 0.014, *p* < 0.001; passive: Est = 0.027, *p* < 0.001), CoP range AP (static: Est = 0.005, *p* = 0.045; unstable: Est = 0.018, *p* < 0.001; passive: Est = 0.020, *p* < 0.001), CoP range ML (static: Est = 0.009, *p* = 0.001; unstable: Est = 0.015, *p* < 0.001; passive: Est = 0.025, *p* < 0.001).

Focusing on the balance*task-type interactions: both area-related measures showed an overall tendency to increase under dual-task conditions, with their absolute values being highest in the passive balance condition, regardless of task type (Figure 3). Despite this shared pattern, the two outcomes differed markedly across task types and balance conditions. Trunk sway area, in the static condition, significantly increased in all tasks requiring an interaction with the screen (RTT and SCWT) with respect to ST, with the SCWT task generating a notably larger increase (post hoc: RTT vs. ST: *p* = 0.016, SCWT 1–3 vs. ST: all *p* < 0.001). In the unstable condition, all dual tasks, independently of the nature of the additional task, led to significant increases in sway compared to ST (ST vs. RTT: *p* = 0.003, ST vs. SS and SCWT 1–3: all *p* < 0.001). In the passive condition, only the dual-tasks involving a cognitive concurrent task caused a significant increase in sway compared to ST (ST vs. SS, ST vs. SCWT 1–3: all *p* < 0.001). Also, in both dynamic conditions, SCWT 3 elicited a greater increase in trunk sway also compared to the RTT (unstable: *p* = 0.021, passive: *p* < 0.001). CoP sway area instead showed more limited sensitivity to dual-tasking: no significant changes were found in the static or passive conditions, and only in the unstable condition, SS produced a significant increase in sway compared to ST (*p* = 0.018).

Regarding the dual-task effect for secondary outcomes, the influence was generally milder than what was observed for the primary measures (Figure 4). For the CoP ranges, both of which showed significant balance*task-type interactions, neither the AP nor ML component exhibited a dual-task effect under static balance. In contrast, effects emerged under dynamic conditions. Specifically, the CoP range in the AP direction increased in the unstable balance condition from the ST to the SS (*p* = 0.010), whereas the CoP range in ML direction increased in SS compared to ST (*p* < 0.001), and this effect was present in both unstable and passive conditions.

Finally, all balance metrics showed that the effect of age on balance performance differed depending on the additional concurrent task (all task-type*age p < 0.050), except the CoP AP range (*p* > 0.050, Table 4). Also, a significant *three-way interaction* was found only for CoP sway area (*p* = 0.001), suggesting that this measure was uniquely sensitive to the combined modulation of balance condition, task complexity, and age. Given the presence of multiple interactions across experimental factors, we conducted targeted post hoc analyses and refitted the models separately for each balance condition to precisely quantify the effects of the concurrent task and age. Results of the model for the different balance conditions are reported in Table 5.

Within each balance condition, the age*task-type interaction was significant for trunk sway area in the unstable condition (*p* = 0.003), and for CoP sway area in both the static (*p* = 0.021) and passive (*p* < 0.001) conditions (see Table 5).

Similarly to the primary outcomes, the secondary measures also demonstrated significant age*task-type interactions that appeared only in specific conditions: trunk range AP showed significant interactions in the static and unstable conditions (both *p* = 0.020), trunk range ML in the static and passive conditions (*p* < 0.001 and *p* = 0.048, respectively), and CoP range ML in the static condition (*p* < 0.001), as shown in Table 5. CoP range AP was the only measure that did not show a significant age*task-type interaction in any of the balance conditions.

For the trunk sway area in the unstable condition, the effect of age increased progressively with task complexity, showing no effect in ST and RTT. Dual-task conditions elicited increasingly larger age-related increases in sway, with the biggest effect for SCWT 3, followed by SS and SCWT 1, whereas SCWT 2 was not significant (see Table 5). Converted estimates indicated annual increases in trunk sway area ranging from approximately 0.005 m^2^/s^4^ (SCWT 1) to 0.23 m^2^/s^4^ (SCWT 3). For CoP sway area, results were similar to those observed for trunk sway: no significant age-related increases were observed for ST or RTT in either static or passive conditions. In both conditions, all cognitively demanding dual tasks showed significant age-related increases, with larger effects in the passive condition and the strongest effect consistently observed for SCWT 3 (static: Est = 0.006, meaning 0.01 cm^2^ per year; passive: Est = 0.047, meaning 0.56 cm^2^ per year).

For the trunk range in the AP direction, age-related effects were limited in the static condition (only SS), but in the unstable condition, both SS and all SCWT tasks showed significant increases (Table 5). For the trunk range in the ML direction, significant age-related increases were observed in static and passive conditions for SS and all SCWT tasks, with RTT significant only in the static condition (Table 5). Across trunk range measures, SCWT 3 consistently showed the largest effect, with annual increases up to 0.05° (AP) and 0.11° (ML). Finally, CoP range in the ML direction showed significant age-related increases only in the static condition, for RTT, SS, and SCWT 1 and 3, with the largest annual increase of 0.02 cm in SCWT 3. All annual changes are reported in the Supplementary Materials.

**Task Duration Affects Only Trunk Metrics in Unstable Conditions**

The duration of the task did not affect balance performance, except for trunk oscillations in the ML direction (*p* = 0.032), where longer task durations were associated with greater trunk oscillations. It is worth noticing that only RTT differed from the standard 30 s duration, ranging from 17.64 to 37.57 s (mean ± std: 27.40 ± 5.68 s) across all balance conditions. A significant duration*balance interaction was found for trunk metrics, but not for CoP metrics, showing an effect of duration on trunk movements in some tasks. Post hoc analyses revealed that task duration significantly increased trunk sway area and trunk ranges only in the unstable balance condition (*p* = 0.006, *p* < 0.001, respectively).

## 4. Discussion

This study investigated how cognitive load, balance conditions, and age interact to influence postural control and cognitive performance across the adult lifespan using a robotic balance platform. Leveraging the platform’s capacity to deliver precise and customizable balance perturbations, we aimed to explore cognitive and balance outcomes that most effectively reveal age-related effects in dual-task conditions of varying complexity, in both static and dynamic balance scenarios.

Most dual-task research has focused on walking combined with a single speech-based cognitive task, which inherently restricts the level of cognitive load that can be imposed. Even studies targeting balance under dual-task conditions typically incorporate only one concurrent cognitive task. In contrast, our approach employed a touchscreen-integrated medical robotic device that enabled diverse assessments across multiple balance conditions, allowing the evaluation of both single balance tasks and a broader range of concurrent dual-task conditions. To include a purely cognitive condition with minimal motor demands beyond maintaining balance, we added the SS task, which relies on sustained attention, working memory, and mental arithmetic [44,45]. To further increase cognitive load, we included a digital version of the SCWT, which engages inhibitory control, selective attention, and cognitive flexibility [46,47], while introducing a motor component due to the touchscreen interaction. Finally, to dissociate cognitive and motor contributions within the Stroop paradigm, we incorporated the RTT as a basic motor task, requiring minimal cognitive processing while using the same touchscreen interaction as the SCWTs.

By integrating multiple concurrent tasks and systematically varying balance conditions, ranging from static to dynamic ones, our study extends the dual-task paradigm. This approach allowed us to isolate cognitive, motor, and age-related influences on postural control and cognitive performance and to identify configurations most sensitive to aging. Moreover, by including dynamic movements, we improved ecological validity, more closely approximating real-world balance challenges and providing insights that are directly relevant to everyday functional mobility.

### 4.1. Cognitive Performance

Cognitive performance in dual-task conditions was strongly modulated by both task complexity and age, with older adults showing greater susceptibility, particularly when tasks imposed higher executive demands, in line with previous findings [48,49].

The pronounced age-related slowing in reaction time observed in the SCWT, especially in its most demanding version (SCWT 3), likely reflects reduced efficiency of attentional regulation and inhibitory mechanisms with advancing age [50,51,52]. Previous behavioural and neuroimaging studies have linked Stroop interference to diminished processing speed and altered recruitment of prefrontal and parietal networks involved in cognitive control [46,47,52,53,54]. Within this framework, the stronger age effects in SCWT relative to RTT indicate that tasks requiring suppression of automatic responses might be more sensitive to age-related changes than simple reaction tasks.

In contrast to reaction time, age-related differences in the number of correct answers emerged only in SCWT conditions, with correct responses remaining relatively stable across SCWT levels. The restriction of age-related differences in number of correct answers to the SCWT suggests that declines become evident primarily under high executive demands, while the relative stability of correct responses across SCWT levels indicates that participants preserved performance by prioritizing correct responses over response speed. Instead, the absence of age effects in the SS task likely reflects its lower executive demands and reliance on overlearned arithmetic skills, which tend to be preserved with aging [55,56].

To contextualize the clinical relevance of these age-related changes, we compared the annual increases in reaction time observed in this study with published minimal detectable change (MDC) values, which represent the smallest change that can be confidently distinguished from measurement error or variability. This comparison provides a practical estimate of how long it would take for age-related decline to become clinically detectable, thereby offering a time-based perspective on the potential clinical relevance of these changes. For RTT, a MDC of 28 ms has been reported [57]. Based on the observed annual slopes, a clinically detectable change would be expected only after more than three decades in the static and passive conditions, whereas under unstable balance conditions, such a change would emerge after approximately two decades. Instead, for the more cognitively demanding SCWT 3, where a MDC of 140 ms has been found [58], a clinically significant change could be detected after roughly 12 years, independently of the balance condition. This proves that cognitively demanding tasks may be inherently more sensitive to age-related decline than simple reaction time measures. From a clinical perspective, this suggests that high-interference tasks like the SCWT 3 could serve as earlier biomarkers for age-related cognitive decline than simpler tasks. While changes in reaction time during the RTT may be masked by high measurement variability for decades, the shorter 12-year window for SCWT 3 detection implies it is a more robust tool for longitudinal monitoring and for evaluating the efficacy of rehabilitative interventions in aging populations. For the number of correct answers, however, MDC values were not available in the literature, limiting the ability to translate observed changes into clinically meaningful terms.

### 4.2. Balance Performance

Our findings showed that within dynamic scenarios, the passive condition consistently produced the largest oscillations at both the trunk and CoP levels. This is likely due to the continuous externally imposed motion of the platform, which, although predictable, is not contingent on the participant’s actions. As a result, anticipatory postural adjustments are less effective in reducing perturbations, leading to greater reliance on compensatory, multi-segmental responses [59,60]. In contrast, the unstable condition preserves sensorimotor coupling between action and sensory feedback, allowing anticipatory strategies to be more effectively integrated into active regulation of the platform [61,62]. Overall, increasing postural demands progressively engage both distal and proximal control mechanisms, requiring coordinated ankle, hip, and trunk involvement for stabilization [63,64].

An interesting finding of this study is that age-related differences varied substantially across balance conditions. While some age-related differences were detectable in static conditions, they became markedly more pronounced during dynamic balance conditions, consistent with prior works [65,66]. Also, trunk- and CoP-based metrics showed distinct sensitivity to aging: trunk measures were most apparent in global metrics like sway area, whereas direction-specific trunk ranges showed more variability and less consistent effects. CoP measures, however, demonstrated age sensitivity across both global and direction specific parameters. Moreover, the balance condition that maximized age effects differed between measures: trunk metrics demonstrated the largest age differences in the unstable condition, as already found in [67], whereas CoP measures exhibited the largest age differences in the passive condition. Under predictable conditions, balance relies primarily on distal adjustment. As instability increases or becomes unpredictable, the hip and the trunk are progressively engaged to stabilize larger body excursions [59,63,68]. Proximal postural control relies more heavily on attentional and executive resources to coordinate multi-segmental adjustments and respond to perturbations, particularly when balance demands are high [26,69,70,71]. When considering cognitive tasks alongside balance conditions, a graded effect of cognitive load on age-related balance changes emerged. We observed that age-related differences in both trunk and CoP sway area appeared only during cognitively demanding dual tasks. Single balance tasks and dual tasks with a simple motor component did not reveal age effects, even if the RTT increased trunk sway area, in agreement with a previous study reporting no age-related differences under simple dual-task conditions with a purely motor concurrent task [72]. This reinforces the idea that cognitive load, more than motor interaction, reveals age-related deficits. These findings differ from a previous study where single-task dynamic balance alone was sufficient to detect age effects [67]. Another work shows that purely motor–motor dual tasks also fail to substantially increase CoP area [73,74] further supporting that cognitive interference is the primary driver of age-related deterioration. This is further supported by our finding that the SCWT, which requires high levels of inhibitory control, produced the most pronounced declines [75]. Indeed, the SCWT engages a broader neural network that competes directly with the resources required for balance regulation [75]. In contrast, the serial subtraction task produced smaller age-related effects, likely due to arithmetic ability being better preserved with aging or because the relatively low task difficulty (as prior research indicates that task difficulty modulates dual-task interference) [55,56,76].

Interestingly, the absence of age effects during tasks involving screen interaction suggests a light-touch effect [72]. Physical interaction may provide additional somatosensory input, enhancing stability and effectively masking age-related deficits that only become apparent when cognitive load demands attention.

To determine the practical usefulness of these results, we consider whether the observed changes exceed measurement variability. For the CoP sway area, the MDC is ~1.4 cm^2^ [77], meaning that annual increases in SS under static conditions would require more than three hundred years to surpass the MDC, making this deterioration meaningless compared with ~4.1 years in the passive condition, and ~2.5 years for SCWT in the passive condition. This contrast has clear clinical implications. Static balance assessments alone was not sensitive enough to track age-related changes over time, because yearly changes are smaller than measurement error. In contrast, the SCWT 3 in passive conditions allows changes to be detected in about 2.5 years. This provides a sensitive way to challenge the postural control system, enabling clinicians to detect age-related decline within a standard follow-up period. This suggests that the clinical value of CoP measures lies less in static assessment and more in their use within cognitively and posturally demanding conditions. Meanwhile, without established MDC values for trunk sway, it remains unclear whether the observed annual changes would be detectable in practice.

## 5. Future Perspective and Limitations

The present findings should be interpreted with caution in light of several methodological considerations. The modest sample size (N = 60) may have limited detection of subtle effects or complex interactions between balance condition, task type, and age. Also, older participants were still relatively young (in their mid-sixties) and physically active, which could have mitigated age-related differences in both balance and cognitive performance, potentially underestimating effect sizes. Task duration varied across conditions, specifically for the RTT task, with longer trials linked to increased trunk oscillations in unstable conditions, suggesting that duration may have contributed to variability in proximal postural outcomes, while CoP measures remained largely unaffected. Future studies should standardize task duration and include older and more heterogeneous populations to better characterize age- and task-related effects on both proximal and distal postural control. By improving our understanding of these effects, this approach could also be extended to clinical populations, potentially revealing subtle deficits in postural and cognitive control when performance is challenged under more demanding and ecologically relevant task conditions.

Regarding cognitive performance, future studies should focus on establishing MDC values for measures of correct responses to enable more complete clinical interpretation. Longitudinal research is also needed to determine whether the 12-year detection window identified in our study can reliably predict the transition from healthy aging to mild cognitive impairment.

For balance performance, research should move beyond static assessments and incorporate dynamic dual-task conditions, which are more sensitive to age-related changes. It is also essential to establish MDC values for trunk-based measures. Although our results show that trunk sway is highly sensitive to aging under unstable conditions, it cannot yet be used reliably for diagnostic purposes until measurement variability is standardized.

## 6. Conclusions

This study systematically evaluated how age, balance condition, and the type of concurrent task influence postural control and cognitive performance during motor–cognitive dual tasks performed on a robotic platform. Understanding the main effects of these factors and their interactions is essential to address the study’s primary aim: identifying cognitive and postural control outcomes that reveal age-related changes in unimpaired adults across static and dynamic conditions.

Results showed that cognitive outcomes were primarily influenced by task complexity and age, with reaction time during the most demanding task particularly sensitive to aging and capable of revealing clinically meaningful changes within just a few years. Balance deficits were modest under static conditions but amplified under dynamic challenges, with trunk sway responding mainly to cognitive load and CoP sway most sensitive to balance perturbations; CoP area changes were found also to exceed minimal detectable thresholds within a short timeframe, whereas secondary outcomes would require decades to show clinically meaningful changes.

Overall, our findings highlight the value of cognitively challenging dual-task paradigms, especially during dynamic balance tasks, as they may allow for the earlier detection of declines in both balance and cognitive functions. This approach reveals subtle motor–cognitive interactions that are not captured by simpler single-task balance assessments and may enable earlier detection of individuals at risk for functional decline. Establishing the profile of motor–cognitive interactions in healthy aging is a necessary first step for differentiating physiological age-related changes from pathological processes in clinical populations, thereby guiding future longitudinal and interventional research. Such insights could facilitate earlier identification of functional impairments and guide the development of targeted interventions aimed at maintaining or restoring motor–cognitive function in clinical populations.

## Figures and Tables

**Table 1:** Complete list of MCDTs performed on the robotic device across both protocols (P1 and P2).

MCDT	SS			RTT			SCWT		
Balance	Static	Unstable	Passive	Static	Unstable	Passive	Static	Unstable	Passive
P1	✓	✓		✓		✓		✓	
P2			✓		✓		✓		✓

**Table 2:** Results from the mixed-effects model showing main effects and interactions of balance condition, age, and task type on cognitive outcomes (Reaction time and Number of correct answers). Corresponding F-values (top) and *p*-values (bottom) for all fixed effects and their interactions are presented. The asterisk * indicates an interaction between factors. For statistically significant effects, both F- and *p*-values are displayed in bold.

Outcome	Balance	Age	Task-Type	Balance * Task-Type	Balance * Age	Task-Type * Age	Balance * Task-Type * Age
Reaction time (ms)	**4.05**	**54.59**	**705.05**	**3.28**	0.50	**27.97**	0.87
	**0.019**	**0.001**	**<0.001**	**0.004**	0.605	**<0.001**	0.515
Number of correct answers	**9.43**	**42.41**	**156.44**	**3.25**	0.27	**7.85**	0.512
	**<0.001**	**<0.001**	**<0.001**	**0.004**	0.762	**<0.001**	0.799

**Table 3:** Main effects of Age and Task-type and their interaction Age*task-type for cognitive outcomes across each balance condition. For main effects and interaction both F-values (top) and *p*-values (bottom) are reported. For statistically significant effects, both F- and *p*-values are displayed in bold. For each Age*task-type significant interaction, the Standardized Estimates (Est) and associated *p*-values are reported. The asterisk * indicates an interaction between factors. “n/a” (not applicable) indicates that the age*type interaction was not significant, and therefore computing standardized estimates was not meaningful, while “-” indicates that the specific outcome was not computable for that task.

Outcome (z-Score)	Balance	Age	Task-Type	Age * Task-Type	RT (Est, *p*)	SS (Est, *p*)	SCWT 1 (Est, *p*)	SCWT 2 (Est, *p*)	SCWT 3 (Est, *p*)
**Reaction time**	Static	**43.30**	**161.90**	**7.52**	0.005	-	**0.018**	**0.021**	**0.036**
		**<0.001**	**<0.001**	**<0.001**	0.286		**<0.001**	**<0.001**	**<0.001**
	Unstable	**28.81**	**207.66**	**5.58**	**0.011**	-	**0.022**	**0.014**	**0.031**
		**<0.001**	**<0.001**	**<0.001**	**0.025**		**<0.001**	**0.009**	**<0.001**
	Passive	**36.80**	**145.15**	**7.39**	<0.001	-	**0.020**	**0.019**	**0.031**
		**<0.001**	**<0.001**	**<0.001**	0.959		**<0.001**	**<0.001**	**<0.001**
**Number of correct answers**	Static	**15.18**	**107.02**	1.41	-	n/a	n/a	n/a	n/a
		**<0.001**	**<0.001**	0.248					
	Unstable	**13.32**	**79.25**	**5.48**	-	−0.004	**−0.032**	**−0.021**	**−0.028**
		**0.001**	**<0.001**	**0.002**		0.542	**<0.001**	**0.006**	**<0.001**
	Passive	**32.57**	**43.74**	**4.06**	-	−0.012	**−0.037**	**−0.031**	**−0.028**
		**<0.001**	**<0.001**	**0.010**		0.068	**<0.001**	**<0.001**	**<0.001**

**Table 4:** Results from the mixed-effects model showing main effects and interactions of balance condition, task-type, age and duration on balance outcomes: primary balance outcomes shown in red and secondary outcomes in blue. Corresponding F-(top) and *p*-values (bottom) for all fixed effects and their interactions are presented. The asterisk * indicates an interaction between factors. For statistically significant effects, both F- and *p*-values are displayed in bold.

Outcome	Balance	Age	Task-Type	Duration	Balance * Task-Type	Balance * Age	Task-Type * Age	Duration * Balance	Balance * Task-Type * Age
Trunk Sway Area (m^2^/s^4^)	**62.08**	**22.79**	**54.11**	2.65	**3.14**	**7.38**	**5.46**	**6.33**	0.985
	**<0.001**	**<0.001**	**<0.001**	0.104	**<0.001**	**<0.001**	**<0.001**	**0.002**	0.455
CoP Sway Area (cm^2^)	**528.34**	**30.58**	**6.20**	2.80	**2.78**	**23.46**	**2.91**	1.73	**2.94**
	**<0.001**	**<0.001**	**<0.001**	0.095	**0.002**	**<0.001**	**0.013**	0.179	**0.001**
Trunk range AP (°)	**53.56**	**12.07**	**27.71**	1.51	1.17	2.10	**3.68**	**7.94**	0.64
	**<0.001**	**0.001**	**<0.001**	0.220	0.074	0.123	**0.003**	**<0.001**	0.773
Trunk range ML (°)	**46.24**	**26.77**	**50.60**	**4.61**	1.52	1.67	**7.53**	**5.05**	0.24
	**<0.001**	**<0.001**	**<0.001**	**0.032**	0.128	0.189	**<0.001**	**0.007**	0.992
CoP range AP (cm)	**558.73**	**43.22**	**14.87**	2.76	**3.78**	**11.40**	1.02	0.25	0.96
	**<0.001**	**<0.001**	**<0.001**	0.097	**<0.001**	**<0.001**	0.404	0.772	0.474
CoP range ML (cm)	**752.31**	**54.04**	**11.57**	2.20	**4.63**	**16.29**	**2.74**	0.86	1.39
	**<0.001**	**<0.001**	**<0.001**	0.138	**<0.001**	**<0.001**	**0.018**	0.413	0.178

**Table 5:** Main effects of Age and Type and their interaction Age*type for balance outcomes across each balance condition. For main effects and interaction both F-values (top) and *p*-values (bottom) are reported. For each Age*type significant interaction, the Standardized Estimates (Est) and associated *p*-values for standardized outcomes are reported. The asterisk * indicates an interaction between factors. For statistically significant effects, both F- and *p*-values are displayed in bold. Balance outcomes include primary measures (in red) and secondary measures (in blue). “n/a” (not applicable) indicates that the age*type interaction was not significant, and therefore computing standardized estimates was not meaningful.

Outcome (z-Score)	Balance	Age	Task-Type	Age * Task-Type	ST (Est, *p*)	RTT (Est, *p*)	SS (Est, *p*)	SCWT 1 (Est, *p*)	SCWT 2 (Est, *p*)	SCWT 3 (Est, *p*)
**Trunk sway area **	Static	3.26	**31.87**	1.50	n/a	n/a	n/a	n/a	n/a	n/a
		0.075	**<0.001**	0.191						
	Unstable	**26.47**	**18.29**	**3.76**	<0.001	0.015	**0.027**	**0.021**	0.016	**0.046**
		**<0.001**	**<0.001**	**0.003**	0.961	0.098	**0.004**	**0.027**	0.090	**<0.001**
	Passive	**13.42**	**13.87**	1.00	n/a	n/a	n/a	n/a	n/a	n/a
		**<0.001**	**<0.001**	0.416						
**CoP sway area**	Static	**24.05**	**6.82**	**2.75**	0.002	0.001	**0.003**	**0.004**	**0.003**	**0.006**
		**<0.001**	**<0.001**	**0.021**	0.092	0.363	**0.004**	**<0.001**	**0.008**	**<0.001**
	Unstable	**25.73**	**10.59**	2.12	n/a	n/a	n/a	n/a	n/a	n/a
		**<0.001**	**<0.001**	0.066						
	Passive	**22.37**	**4.73**	**5.02**	0.004	0.015	**0.030**	**0.037**	**0.038**	**0.047**
		**<0.001**	**<0.001**	**<0.001**	0.557	0.135	**0.002**	**<0.001**	**<0.001**	**<0.001**
**Trunk range AP**	Static	1.62	**15.62**	**2.75**	−0.001	-0.002	**0.030**	0.004	<0.001	0.003
		0.207	**0.007**	**0.020**	0.835	0.818	**<0.001**	0.659	0.951	0.684
	Unstable	**12.70**	**14.50**	**2.77**	0.006	0.012	**0.027**	**0.025**	**0.017**	**0.029**
		**<0.001**	**<0.001**	**0.020**	0.301	0.118	**<0.001**	**0.001**	**0.026**	**<0.001**
	Passive	**7.27**	**6.79**	0.52	n/a	n/a	n/a	n/a	n/a	n/a
		**0.009**	**<0.001**	0.759						
**Trunk range ML**	Static	**22.77**	**35.09**	**4.53**	<0.001	**0.015**	**0.022**	**0.021**	**0.014**	**0.025**
		**<0.001**	**<0.001**	**<0.001**	0.970	**0.028**	**<0.001**	**<0.001**	**0.021**	**<0.001**
	Unstable	**26.47**	**21.81**	2.05	n/a	n/a	n/a	n/a	n/a	n/a
		**<0.001**	**<0.001**	0.094						
	Passive	**17.05**	**10.28**	**2.29**	0.008	0.015	**0.034**	**0.035**	**0.034**	**0.035**
		**<0.001**	**<0.001**	**0.048**	0.284	0.179	**0.001**	**<0.001**	**<0.001**	**<0.001**
**CoP range AP**	Static	**7.84**	**3.69**	0.41	n/a	n/a	n/a	n/a	n/a	n/a
		**0.003**	**<0.001**	0.838						
	Unstable	**25.34**	**9.19**	1.11	n/a	n/a	n/a	n/a	n/a	n/a
		**<0.001**	**<0.001**	0.356						
	Passive	**28.43**	**11.23**	2.09	n/a	n/a	n/a	n/a	n/a	n/a
		**<0.001**	**<0.001**	0.070						
**CoP range ML**	Static	**25.56**	**10.95**	**5.33**	0.002	**0.013**	**0.023**	**0.022**	0.009	**0.024**
		**<0.001**	**<0.001**	**<0.001**	0.685	**0.033**	**<0.001**	**<0.001**	0.077	**<0.001**
	Unstable	**35.92**	**12.09**	0.54	n/a	n/a	n/a	n/a	n/a	n/a
		**<0.001**	**<0.001**	0.741						
	Passive	**33.43**	**6.17**	2.13	n/a	n/a	n/a	n/a	n/a	n/a
		**<0.001**	**<0.001**	0.065						

## Data Availability

The data presented in this study are available on request from the corresponding authors.

## Supplementary Materials

The following supporting information can be downloaded at: https://www.mdpi.com/article/10.3390/s26061847/s1, File S1: Additional analysis and data table.

## Abbreviations

The following abbreviations are used in this manuscript:

ST	Single-task
DT	Dual-task
MCDT	Motor–cognitive dual-task
CoP	Center of pressure
AP	Anteroposterior
ML	Mediolateral
RTT	Reaction time test
SS	Serial Subtraction
SCWT	Stroop Color and Word Test
