# Supplementary material for: Age-Related Differences in Cognitive and Postural Performance During Dynamic Dual-Tasks
Source: Sensors (Basel). 2026 Mar 15;26(6):1847. doi: 10.3390/s26061847 (PMC13030157; doi:10.3390/s26061847)
Supplement: Supplementary file 1 [file sensors-26-01847-s001.zip › sensors-4173887-supplementary.pdf]

Supplementary materials

Assessment of baseline differences in primary balance outcomes

Before focusing on our main analysis as mentioned in the main text, we first verified that the two protocol groups did not differ in the primary balance outcomes at baseline. A general linear model was employed with balance outcome as the dependent variable, protocol as the fixed factor, and age as a covariate. The effect of protocol was not significant for either Trunk Area (p = 0.125) or CoP Area (p = 0.233), indicating comparable baseline performance between the two groups in the standing position.

Annual changes for cognitive and balance outcomes

For completeness, we include the table below, which presents the annual changes for each cognitive and balance outcome in the balance conditions where the age*task-type interaction was significant. Annual changes were calculated by multiplying the standardized beta estimates, representing change in standard deviations per year, by the standard deviation of each outcome variable, thereby converting the effects into the original measurement units. In the table, each outcome and task reports the estimated annual change in the units indicated in the outcome column, corresponding to the change per year of age.

Outcome	Balance	ST	RTT	SS	SCWT 1	SCWT 2	SCWT 3
Reaction time (ms)	Static	-	0.657	-	**2.751**	**3.567**	**11.306**
	Unstable	-	**1.393**	-	**4.449**	**2.298**	**8.750**
	Passive	-	0.113	-	**3.428**	**3.248**	**8.405**
Number of correct answers	Unstable	-	-	-0.001	**-0.142**	**-0.082**	**-0.115**
	Passive	-	-	-0.002	**-0.177**	**-0.136**	**-0.116**
Trunk Sway Area (m^2/s^4)	Unstable	< 0.001	0.005	**0.011**	**0.006**	0.004	**0.023**
CoP Sway Area (cm^2)	Static	0.001	0.001	**0.003**	**0.007**	**0.004**	**0.011**
	Passive	0.038	0.168	**0.339**	**0.386**	**0.417**	**0.560**
Trunk Range AP (°)	Static	< 0.001	0.003	**0.0412**	0.005	0.001	0.004
	Unstable	0.005	0.0178	**0.0449**	**0.0296**	**0.020**	**0.0463**
Trunk Range ML (°)	Static	< 0.001	**0.023**	**0.037**	**0.034**	**0.020**	**0.048**
	Passive	0.010	0.028	**0.114**	**0.117**	0.094	**0.113**
CoP Range ML (cm)	Static	< 0.001	0.008	**0.015**	**0.012**	0.005	**0.015**
